# Deep multiple instance learning for foreground speech localization in ambient audio from wearable devices

**DOI:** 10.1186/s13636-020-00194-0

**Published:** 2021-02-03

**Authors:** Rajat Hebbar, Pavlos Papadopoulos, Ramon Reyes, Alexander F. Danvers, Angelina J. Polsinelli, Suzanne A. Moseley, David A. Sbarra, Matthias R. Mehl, Shrikanth Narayanan

**Affiliations:** 1 Signal Analysis and Interpretation Laboratory, University of Southern California, Los Angeles, US; 2 Department of Psychology, University of Arizona, Tuscon, US; 3 Department of Neurology, Indiana University School of Medicine, Indianapolis, US; 4 Department of Psychology, MN Epilepsy Group, St. Paul, US

**Keywords:** Foreground speech detection, Multiple instance learning, Wearable audio, Weakly labeled audio

## Abstract

Over the recent years, machine learning techniques have been employed to produce state-of-the-art results in several audio related tasks. The success of these approaches has been largely due to access to large amounts of open-source datasets and enhancement of computational resources. However, a shortcoming of these methods is that they often fail to generalize well to tasks from real life scenarios, due to domain mismatch. One such task is foreground speech detection from wearable audio devices. Several interfering factors such as dynamically varying environmental conditions, including background speakers, TV, or radio audio, render foreground speech detection to be a challenging task. Moreover, obtaining precise moment-to-moment annotations of audio streams for analysis and model training is also time-consuming and costly. In this work, we use multiple instance learning (MIL) to facilitate development of such models using annotations available at a lower time-resolution (coarsely labeled). We show how MIL can be applied to localize foreground speech in coarsely labeled audio and show both bag-level and instance-level results. We also study different pooling methods and how they can be adapted to densely distributed events as observed in our application. Finally, we show improvements using speech activity detection embeddings as features for foreground detection.

## Introduction

Wearable devices are used widely in a variety of health and lifestyle related applications, from tracking personal fitness to monitoring patients suffering from physical and mental ailments. Advances in wearable materials and sensing technology have facilitated steady increase in the use of such devices by making them unobtrusive, inexpensive and more reliable [1, 2].

Audio is an essential stream of information that can be measured in addition to the various physiological signals (e.g., ECG, EEG) via such devices. Audio signal can provide important cues about a person’s environment, their speech communication, and social interaction patterns [3]. Quantity and quality of communication and social interactions have been shown to be linked to a person’s well-being, happiness, and overall sense of life satisfaction [4, 5]. Moreover, it has been shown that speech rate [6] and vocal prosody [7] are strong indicators of depression severity in patients. As a result, multiple wearable technologies aimed at obtaining unobtrusive audio recordings in natural, non-laboratory real-world conditions have been proposed [8–10]. In such an egocentric setting, we are typically interested in detecting and analyzing speech uttered by the participant wearing the device, which is commonly referred to as foreground speech [11].

One of the major challenges in processing audio from a wearable device is dealing with the varying ambient noise conditions. Since the participant is not restricted to any particular audio environment, detecting audio-related events in varying acoustic conditions is extremely challenging [12]. A specific issue is the interference of speech from background speakers with the foreground speech. Typical voice activity detection systems are not designed to distinguishing between different speaker characteristics to make this distinction. Additionally, the egocentric devices across participants will rarely be identical in nature (i.e., frequency responses of these devices vary), resulting in non-uniform channel conditions across devices. For all of the above reasons and more, performance of foreground detection systems designed for clean environments deteriorates in real-world operating conditions [11].

Data-driven neural network-based models have proven to be effective for such classification tasks, given large amounts of labeled data. With the increased deployment of microphone recorders in mobile and other IoT devices, including voice speakers, alongside the widespread use of voice agents such as Siri and Alexa, collection of audio data at scale has become inexpensive, resulting in large amounts of audio of interaction of people in, and of, their environments.

However, obtaining annotations for these data at scale is often cumbersome, expensive, and can be prone to human errors. Furthermore, since most audio systems are developed to operate at frame-level (10 ms duration typically), annotations at that scale may be necessary in order to train supervised models for the task at hand. One way of bridging this gap is to obtain coarse labels at a lower time-resolution (30 s segments in our case) and leverage machine learning techniques such as multiple instance learning (MIL) to model the task at segment-level and further interpolate the results at frame level. This particular concept known as localization involves finding regions of interest within a data record. For example, finding objects of interest in an image [13] or, more specifically in our case, detecting occurrence of particular events in coarsely labeled audio clips [14]. Since we focus here on temporal localization and not spatial, it is important to distinguish our work from audio localization techniques involving direction of arrival estimation in multi-channel audio signals [15].

**Fig. 1:**
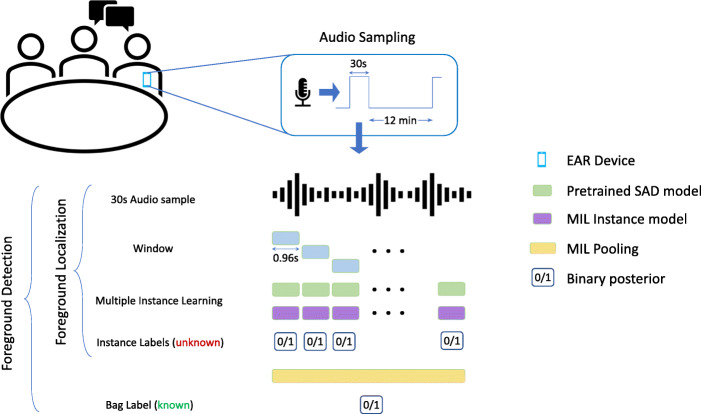
MIL pipeline. Learning to detect and localize foreground speech in wearable-device audio

In this work, we propose a method for localizing foreground speech within audio clips using multiple instance learning. The pipeline of our approach is shown in Fig. 1. We use audio collected from mobile EAR devices [8], consisting of 90K recording clips of 30 s duration, from over 200 participants. We use non-overlapping windows of 0.96 s duration to create instances from each 30 s audio clip (bag). We then use time-distributed deep neural network architectures to model the MIL framework, which also helps reduce the number of parameters and computation cost to process long utterances (30 s duration). We test several MIL pooling methods such as max and average pooling as well as attention-based pooling for obtaining segment level predictions from frame-level posteriors. Furthermore, we show further improvement using embeddings from speech activity detection task transfer-learned for foreground detection.

## Related work

### Foreground detection

Foreground (FG) speech detection is the task of detecting regions in an audio recording where the person of interest (typically wearing the recording device) is speaking. FG detection has been studied in the literature for an array of mobile-device applications. For example, Dehak et al. showed that zero-frequency filtered signals (ZFFS) can be used to reliably detect foreground speech in the presence of noise and background speech [16]. However, the method faces challenges in cases where the background speaker is close to the device since ZFFS exploits difference in nature of speech and noise and does not capture foreground speech related characteristics. More recently, convolutional neural networks have been used to train a FG detector on an open-domain meeting dataset [11]. The target dataset was collected during meetings from participants with wearable recorders on their person. It was shown that fine-tuning of the model on the target dataset was required to obtain best FG detection results.

### Multiple instance learning

MIL is a *weakly supervised* machine learning algorithm wherein each individual training sample, called a *bag*, is organized as a collection of samples, called *instances*. Instances exhibit similar properties as a bag and can be assigned the same set of labels as a bag. Under the standard assumption of MIL, a bag is labeled positive if at least one of the instances belonging to the bag is positive and negative if all of the instances are negative. For training, it is assumed that labels are available for each bag, but not for the individual instances of a bag, hence the term weakly supervised.

One of the more successful applications of MIL has been that of object detection in images [13, 17, 18]. Here, each image is treated as a bag, and sub-regions of the image are its instances. Naturally, the presence of an object in a sub-region of an image implies its presence in the image (i.e, follows standard assumption). The chief utility of MIL is in being able to *localize* objects via positive instances, in addition to detecting the presence/absence of objects in an image. MIL in the context of neural networks has been extensively reviewed for a variety of image applications [18].

Multiple instance learning (MIL) for the purposes of classifying coarsely labeled audio has been primarily studied for tasks such as audio event detection [19–21]. These approaches have been formulated as multi-class event detection using audio data labeled at coarse segments (≥ 10 s). Segments are then typically split into uniform sub-segments which are treated as instances and MIL is used for event detection at bag level.

There are two major distinctions in the application of MIL in our proposed work. First, the density of FG labels is much higher compared to audio events, i.e., a single bag consisting of 100 instances could have up to 100 positive FG samples, whereas a single audio event being detected 100 times in a bag is much less likely due to the sparse distribution of audio events across time. Second, these approaches evaluate their methods only at the bag-level, whereas our proposed method attempts to use MIL to localize the event at the instance level and evaluate it as such. MIL for localization in audio has been studied for the tasks of phoneme recognition and sound event detection. Due to difference in nature of occurrence, the pooling function used plays a major role in modeling such events. For example, in [14], two pooling methods were used: max pooling and noisy-or pooling. The authors found that max pooling was better suited for localization since noisy or pooling resulted in spiky and low-amplitude posteriors for positive instance samples due to multiplicative nature of the pooling function.

### Transfer learning

Transfer learning is a machine learning concept that is used to learn rich feature representations from a source task and subsequently apply them to a target task, with the constraint that both the tasks be strongly related [22]. This approach is usually adopted primarily for its relative ease of training source task as against target task owing to larger dataset size, ease of procuring labels, etc. Additionally, compressed representations are more light-weight as compared to raw features and can reduce training complexity.

A few examples of transfer learning applications include transferring knowledge from speech recognition system trained on one language to another (e.g., English to German) [23] and using embeddings from a large-scale audio event classification task (over 1 million 10 s audio clips) to improve gender identification [24]. In the context of this work, we aim to use feature embeddings learnt from speech activity detection task for foreground detection.

In our approach, we leverage benefits of the aforementioned aspect of having larger amounts of labeled data available to train speech activity detection systems and hence get richer embeddings for foreground detection. Furthermore, since we deal with long audio segments, using raw frame-level features can prove to be computationally expensive as compared to using highly compressed feature-embeddings.

## Multiple instance learning

In this section, we discuss the MIL formulation as it is applied to detect foreground speech in coarsely labeled audio. We also describe the methodology to localize foreground speech at frame level.

### MIL formulation

In the context of this work, MIL can be formulated as learning to detect regions of foreground speech within an audio segment. In this case, a single segment (bag) of duration T is split into N consecutive non-overlapping sub-segments (instances) of duration *t*=*T*/*N*. Specifically, consider an audio segment *x* constructed in this fashion: *x*=[*x*_1_,*x*_2_,...,*x*_*N*_] and its corresponding label *y*_*x*_={0,1} to indicate absence/presence of foreground speech in the duration of *x*. Then, instance-label pairs (*x*_1_,*y*_1_), (*x*_2_,*y*_2_),... (*x*_*N*_,*y*_*N*_) can be constructed, where *y*_*i*_ correspond to sub-segments *x*_*i*_ respectively. In our application of MIL, the label *y* that corresponds to segment *x* is *y*=1 if at least one of the *y*_*i*_,*i*=1,2,...,*N* is one.

1 $$ y = y_{1} \vee y_{2} \vee \ldots \vee y_{N} ; \; y_{i} =\{0,1\}, \; i=1,2,\ldots N  $$

where ∨ stands for the logic operator *OR*. Whenever the instance labels *y*_*i*_ can be expressed as posterior probabilities (e.g. they are the outputs of probabilistic models), we get the typical MIL formulation:

2 $$ y = \max{(y_{i})}; \; y_{i} =[0,1], \; i=1,2,\ldots N  $$

### Pooling methods

A pooling layer is implemented to aggregate the posteriors from instances. Several pooling methods have been proposed for neural network-based MIL approaches [14, 18]. These can be broadly classified into two categories, namely, embedding and instance-based pooling. For the purposes of interpretability and localization, we focus only on instance based pooling methods. Due to the nature of the MIL framework (eqn 2), max pooling is an obvious choice as a pooling function. Average pooling has also been shown to work well in such a framework [18].

Although traditional attention-based pooling is designed for the embedding scenario [18], they can be generalized to the instance based approach (Table 1). Additionally, the attention-weight activation can be modified to account for more densely distributed labels such as FG. Since softmax activation constrains the sum of the weights to 1, it is not necessarily appropriate in the scenario where the model needs to attend on a large portion of the audio segment. Hence, a sigmoid-activation attention layer is introduced in order to be able to attend to each of the instances independently, while maintaining the dot-product attention. Note that in this case, we would need an additional scaling factor for computing the bag-label. Finally, a hybrid attention + max-pool model is proposed wherein the dot-product pooling operation is replaced by max pooling, but the attention mechanism is maintained.

**Table 1:** Different MIL pooling methods for FG localization where, $\bar {y} = [y_{1}, y_{2},..., y_{N}]$

Pooling method	Pooling operation	Instance label
Max pooling	*y*=*max*(*y*_*i*_)	*y*_*i*_
Average pooling	$y = 1/N \sum _{i} y_{i}$	*y*_*i*_
Attention (softmax)	$y = \sum _{i} a_{i} y_{i} a_{i} = softmax(w^{T} f(\bar {y})) f(\bar {y}) = tanh(V \bar {y}^{T})\odot sigmoid(U \bar {y}^{T})$	*a*_*i*_*y*_*i*_
Attention (sigmoid)	$y = 1/N \sum _{i} a_{i} y_{i} a_{i} = sigmoid(w^{T} f(\bar {y}))$	*a*_*i*_*y*_*i*_
Hybrid (attention + max pooling)	$y = max (a_{i} y_{i}) a_{i} = sigmoid(w^{T} f(\bar {y}))$	*a*_*i*_*y*_*i*_

## Dataset

For the purpose of audio collection, we use the mobile-EAR device [8]. We analyze data collected from two broad categories of participants, which we call EAR Aging Study (AS) and Ear Divorce Study (DS).

AS data were collected over a period of 2 years, from 93 participants in the age group of 65–90 years [25]. DS, on the other hand, consists of data from 122 individuals who have been through a divorce [26]. These data were collected over a 5-year period (Table 2).

**Table 2:** Dataset description

Dataset	Number of participants	Number of samples	% with foreground	Train/Val/Test (speakers)
AS	93	33363	21.5	75 / 9 / 9
DS	122	56091	29.7	98 / 12 / 12

The audio signal for each participant was split into 30 s chunks for the purposes of annotation. Each of these 30 s clips was then annotated for a broad number of categories that describe the ambient setting, presence of social group, nature of interaction, and other audio cues such as emotional state of the participant. The Aging Study was annotated by a single human coder, while the Divorce Study was annotated by two different coders (for all clips).

In this work, we focus on the foreground speech label. This binary label is annotated as 1 if the person wearing the device speaks at any point during the 30 s duration of the file. In the case of the Divorce Study with 2 annotations per clip, disagreement (< 1000 clips) between annotators was resolved by randomly picking one of the two annotations.

Each of the AS/DS datasets are split into train, validation, and test splits in the ratio of 80:10:10. For fair evaluation of our proposed methods, we ensure that the participants are non-overlapping across the splits, i.e., any participant’s device audio in the test set will not be seen in either of the training or validation sets. We use the training set to update model parameters, validation set to determine early stopping, and finally the test set to show the performance of our method.

## Experiments

In this section, we discuss the setup for the experiments conducted, such as the neural network architectures and features used for the task of foreground detection/localization. For each of the experiments, we choose the value of *t*=0.64*s*(*T*=30*s*) for convenience.

### Neural network architectures

We performed both architecture search as well as hyper-parameter tuning for determining the best-performing model architecture and the number of convolutional blocks and hidden layer dimensions for recurrent and fully connected layers therein.

The CNN-based architectures include standard CNN, CNN-GAP, CLDNN, and CNN-TD models [27]. The difference in these architectures is in the handling of the final output of the convolutional layers. In the standard CNN, the output is flattened and fed into a dense layer, while in CNN-GAP, a 2D global average pooling layer is used to condense the output of the convolutional block. For the CLDNN and CNN-TD architectures, the temporal dimension of the convolutional output is retained. The frequency and filter-channel dimensions are merged and fed into each of these, the outputs of which are then pooled temporally. CNN-TD uses two time-distributed fully connected layers which share their weights across the pseudo-temporal dimension, while CLDNN uses LSTM and bi-LSTM layers to process the temporal embeddings.

We also implement one and two layer LSTM and biLSTM architectures. We exclude some of the heavier architectures from our search for the baseline models, due to resource constraints and high input dimension of our features (3000 x 64 in the case of log-Mel-based approach). We picked the best performing model on the validation set and report results on the held-out test set.

### Features for foreground detection

We develop neural-network models for the binary task of foreground speech detection in a 30 s segment. As baseline features, we use 64-dimensional log-Mel filterbanks extracted at 10 ms duration using *Kaldi*^1^. The 3000 x 64-dimensional features are then reduced to binary class posteriors for foreground classification.

Embeddings from a speech activity detection model trained on movie data [27] are used for the purposes of transfer learning for foreground detection task. Convolutional neural network models were trained on 0.64 s duration audio segments for a two class speech/non-speech classification problem. In this work, we use the 256-dimensional global average pooling layer outputs from the CNN-GAP flavor of the models since it has been shown to attend to speech regions in the log-Mel spectrogram. Extracting these embeddings from non-overlapping segments results in 45 x 256-dimensional features as inputs to our models.

### Foreground localization

**Fig. 2:**
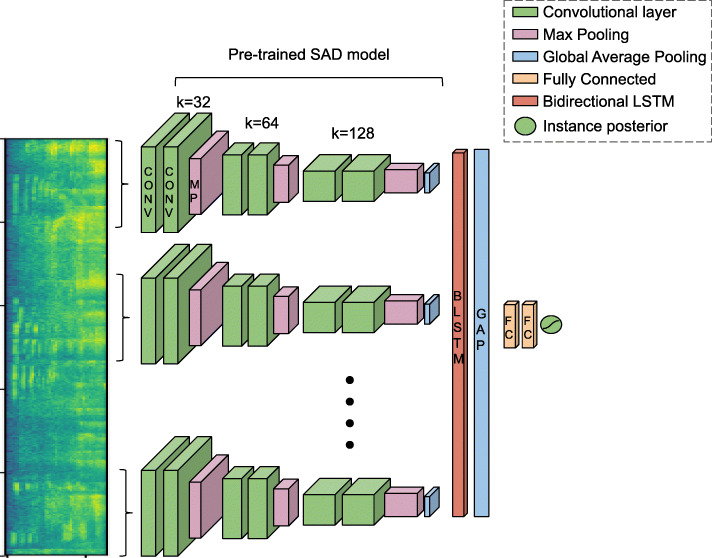
MIL model. Multiple instance learning framework for foreground detection

We then trained MIL models following the framework described in Section 3. Time-distributed fully connected models were trained using SAD embeddings as shown in Fig. 2. Five different pooling methods (Section 3.2) were evaluated at both segment and frame level.

For the purposes of evaluating the localization approaches, we use gentle^2^ to obtain frame-level labels for foreground speaker. Gentle is an open-source robust forced-alignment tool which can be used to robustly align a text transcript with its corresponding audio. Since we have manually transcribed text for foreground speech, we use these to gentle-align to raw audio at word-level within the test set. We only perform gentle-alignment on utterances without any identifying information about the participant. Post alignment, we pick utterances with at least 90% successfully aligned words, in order to have high confidence in our evaluation labels. We use these words as the positive class (FG) labels for our evaluation. For the negative class, we retain the original negative-samples (no FG) for the segment-level evaluation, since a negative-labeled bag implies that each the sub-segments are also negative. Due to high class imbalance, we report the percentage speech detected at a fixed false alarm rate of 1%.

## Results and discussion

As a baseline for the bag-level approaches, we show results using the CNN-based *Vggish slimmer* [11] model trained on ICSI meeting data (Table 3). Since this model is trained on frame-level (10 ms duration) features, we use the max operation to aggregate frame-level posteriors to obtain utterance predictions.

**Table 3:** Foreground detection results on utterance (bag) level approaches

	Divorce study				Aging study			
	Precision	Recall	F1 score	AUC	Precision	Recall	F1 score	AUC
VGGish slimmer [11]	0.34	0.96	0.5	0.71	0.38	0.94	0.54	0.66
Log-Mels	0.78	0.68	0.72	0.8	0.67	0.73	0.7	0.81
SAD embeddings	0.82	**0.82**	**0.82**	**0.87**	**0.77**	**0.81**	**0.79**	**0.87**
MIL (Log-Mels)	0.71	0.7	0.7	0.79	0.64	0.66	0.65	0.77
MIL (SAD emb)	0.83*	0.79	0.81	0.86	0.66	0.83	0.73	0.85

*McNemar’s Test, *p* ≪0.01

While the baseline model detects most foreground utterances (high recall), it predicts high number of false positives resulting in low precision. One reason for this could be domain mismatch between training data and the EAR data. Due to resource constraints and large number of parameters of the model, we do not perform domain adaptation.

For the log-Mels based models, CLDNN architectures give best results on both the datasets. However, models trained on SAD embeddings significantly outperform the log-Mel approaches. The best-performing models on both datasets are bidirectional LSTM models, achieving F1 scores of 0.82 and 0.79 on DS and AS, respectively. For both sets of features, the results of the non-MIL models are *not significantly*^3^ better than MIL models.

It is important to note that the MIL-based models are under-parametrized in comparison to non-MIL models. Also, the non-MIL based methods can leverage 30 s context to make a binary decision while MIL models only have access to instance-level posteriors to make a bag-level decision. For these reasons, we expect the non-MIL models to be an empirical upper-bound to the bag-level performance of the MIL models.

**Fig. 3:**
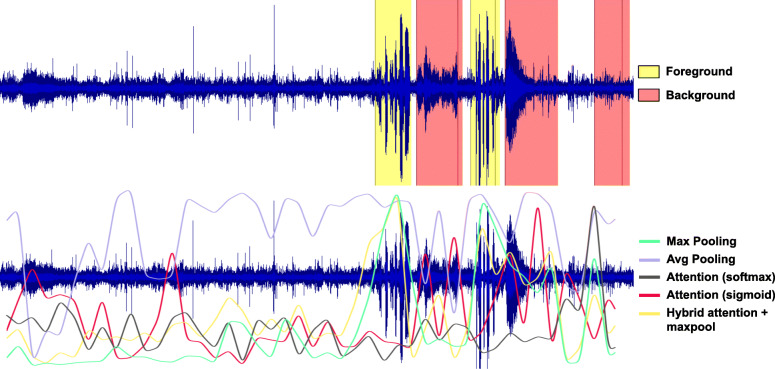
Visualization of different pooling methods for foreground localization using gentle-aligned regions as labels; *top*: ground-truth regions of foreground and background speech; *bottom*: foreground posteriors of different models

The results of the foreground localization using different MIL-based pooling techniques are tabulated in Table 4. Among the non-parametric pooling methods, max pooling outperforms average pooling as expected. Even though average pooling shows good results, from Fig. 3, we can clearly see that it fails to localize the foreground speech effectively. Since the two class labels for the localization are from positive and negative bags respectively, the performance can be explained by the fact that average pooling is learning to predict bag-level labels for each of the instances.

**Table 4:** Foreground localization results on frame (instance) level approaches on DS

MIL model	% speech detected at 1% FAR	Bag-level F1 score
Max pooling	93.7	0.76
Average pooling	88.0	0.74
Attention (softmax)	12.7	0.78
Attention (sigmoid)	68.5	0.78
Hybrid	90.1	0.73

While the softmax-based attention model results in good bag-level performance, it fails miserably in the localization task. This is to be expected since softmax activation is not well-suited to our case where FG labels are densely distributed. The sigmoid-based attention model, however, significantly outperforms the softmax-attention model. This can be attributed to the fact that sigmoid attention treats the instances independently, allowing the network to attend to possibly multiple instances within a segment. Finally, the hybrid model performs significantly better than the purely attention-based pooling approaches. However, it performs only slightly worse than simple max pooling, suggesting that learning of attention weights does not improve performance of localization task.

## Conclusion

In this work, we used multiple instance learning to localize densely distributed events of foreground speech in a coarsely labeled setting. We evaluated MIL models trained at bag-level and showed comparable to results to the best performing neural network architecture. We studied the performance of different pooling methods and introduced two new pooling approaches to improve performance of parametric methods. Future work includes obtaining frame-level annotations for better evaluation of localization experiments as well as detailed error analysis.

## Data Availability

The datasets generated and/or analyzed during the current study are not publicly available due to privacy requirements but are available from the corresponding author on reasonable request.

## Footnotes

1 http://kaldi-asr.org/

2 https://github.com/lowerquality/gentle

3 McNemar’s test, *p* ≪0.01
